# Microstructure and Cross-Sectional Shape of Limb Bones in Great Horned Owls and Red-Tailed Hawks: How Do These Features Relate to Differences in Flight and Hunting Behavior?

**DOI:** 10.1371/journal.pone.0106094

**Published:** 2014-08-27

**Authors:** Crystal A. Marelli, Erin L. R. Simons

**Affiliations:** 1 Arizona College of Osteopathic Medicine, Midwestern University, Glendale, Arizona, United States of America; 2 Department of Anatomy, Arizona College of Osteopathic Medicine, Midwestern University, Glendale, Arizona, United States of America; University of Utah, United States of America

## Abstract

The Red-tailed Hawk and Great Horned Owl are two species of raptor that are similar in body size, diet, and habitat. Both species use their hindlimbs during hunting, but differ in foot morphology, how they approach and immobilize prey, and the average size of prey captured. They also differ in primary flight style: the Red-tailed Hawk uses static soaring and the Great Horned Owl uses flap-gliding. The objectives of this study were to characterize the microstructure and cross-sectional shape of limb bones of these species and examine the relationship with flight and hunting behaviors. The mid-shaft of six limb bones from six individuals of each species was sampled. The degree of bone laminarity (proportion of circular primary vascular canals) and cross-sectional geometric parameters were calculated. In both species, the humerus and femur exhibited features that suggest high resistance to torsional loading, whereas the tibiotarsus and phalanges had a shape more likely to resist compression and bending in a specific plane. The femur of the Red-tailed Hawk exhibited higher laminarity and larger polar moment of area than that of the Great Horned Owl. The tibiotarsus was more elliptical than that of the Great Horned Owl. The hawk approaches prey from a more horizontal axis, takes prey of greater mass, and is more likely to pursue prey on the ground, which could potentially be causing more torsional loads on the femur and bending loads on the tibiotarsus. In addition, differences in polar moment of area of the phalanges between the species could relate to differences in foot morphology or digit length. The humerus and ulna of the flap-gliding Great Horned Owl are more elliptical than the static soaring Red-tailed Hawk, a shape that may better resist the bending loads associated with a larger amount of flapping.

## Introduction

The Red-tailed Hawk (RTH, *Buteo jamaicensis*) and Great Horned Owl (GHO, *Bubo virginianus*) are two widespread species of raptor from distinct distantly related avian groups (Accipitridae and Strigiformes) found within the well-supported land bird clade [1], [2]. The two species often occupy overlapping habitats and are similar in average mass (GHO: 1354 g and RTH: 1126 g) [3], [4]. Red-tailed Hawks and Great Horned Owls are both sit-and-wait predators that prey on primarily mammals and birds, but at different times of day [5], [6]. Although often referred to as diurnal-nocturnal ecological counterparts, studies have shown that the two species actually specialize on different types of prey [5], [6] The Red-tailed Hawk has more dietary diversity, which includes reptiles, and takes prey of significantly greater mass [6].

Both species rely on speed and strength of their hindlimbs to capture prey, but previous research has identified several differences in both hindlimb morphology and specific hunting behaviors between the two species [7], [8]. First, the morphology differs in that the species have different foot morphology and relative robustness of skeletal elements. Red-tailed Hawks are anisodactylous, digit one (the hallux) is directed posteriorly and digits two, three, and four are directed anteriorly [9]. Great Horned Owls are zygodactylous, meaning that digits two and three are directed anteriorly and digits one and four are directed posteriorly [9]. The hindlimb bones of owls are more robust than those of hawks. Owls have shorter and more robust tarsometatarsi relative to other raptors [9], [10]. A shorter tarsometatarsus increases the force produced by the main hindlimb muscles used to flex the tarsometatarsus and digits [11]. Secondly, the two species differ in how prey is approached and immobilized. The Great Horned Owl specializes in small prey and hunts primarily from a perch. The owl vertically descends on prey and uses high force grip to constrict and immobilize prey [9], [10], [12]. Since owls hunt in low light conditions, if the prey escapes the bird will often return to the perch to re-locate the prey [9]. Red-tailed Hawks hunt from both soaring and perched positions. They approach prey from a horizontal axis with hindlimbs extended, use their large hallux to grip prey and often bend their body forward to assist in pinning the prey to the ground [12]–[14]. Hawks are more likely to pursue escaped prey on the ground or use their large talons on digits one and two to drag prey to a new location [10], [15].

The Great Horned Owl and the Red-tailed Hawk also utilize different primary flight modes. Red-tailed Hawks use static soaring, an energy-conserving flight mode in which the bird uses moving air currents to maintain altitude without flapping [8], [16]. In contrast, the Great Horned Owl uses alternating flapping and gliding as its primary flight mode [4]. Despite these differences, the aspect ratio (a measurement of wing length to breadth) between the Red-tailed Hawk and Great Horned Owl is very similar (RTH AR = 5.61, GHO AR = 5.3), indicating a similar overall wing shape between the species [4].

During locomotion, skeletal elements are subjected to a variety of mechanical loads. The use of *in vivo* strain gauges can give an idea of what types of loads a bone is experiencing during different behaviors. One study has shown that the proximal avian forelimb element, the humerus, experiences both torsion (twisting around a neutral axis) and bending loads imparted by the aerodynamic forces on the wing during flapping flight [17]. Also, avian hindlimb elements (tibiotarsus and femur) have been shown to experience bending, axial compression, and torsion, with the femur typically showing predominant torsional strains [18], [19]. However, the amount of each of these types of load experienced by a long bone depends on multiple factors, including locomotor mechanics, body size, and posture. In general, a bone’s ability to resist different mechanical loads is affected by both the bone cross-sectional shape and the bone microstructure.

### Cross-sectional geometry

Cross-sectional geometric parameters, derived from Euler-Bernoulli’s beam theory, describe the amount and distribution of cortical bone in a cross section and may reflect a bone’s resistance to mechanical loadings such as torsion, bending, and axial compression [20], [21]. The second moment of area (I) can be used to characterize the resistance of a bone to bending around an axis [22]. A ratio of the second moment of area in the maximum direction (I_max_) to the second moment of area in the minimum direction (I_min_) can be used to quantify cross-sectional shape, more specifically how circular or elliptical a cross section is. An elliptical cross-section in interpreted to better resist bending loads in a specific direction. The polar moment of area (J) is the sum of I_max_ and I_min_ and both describes the bone’s ability to resist torsional loads and is a measure of the overall resistance to bending [23]. Cortical area (CA) is the amount of cortical bone in a cross section and can be used to estimate a bone’s ability to withstand axial compression [24]. Therefore, according to beam theory: a bone with an elliptically shaped cross section is optimized to resist bending loads in a specific plane, a bone with a circular cross section and high polar moment of area is optimized to resist torsional loads, and a bone with a high proportion of cortical area is optimized to resist axial compression.

Avian long bones have been shown to experience certain loads during specific behaviors [17]–[19], but not all birds necessarily use the same primary locomotory behaviors, so several subsequent studies have investigated how the cross-sectional geometry of limb bones can be used to estimate the different loads placed on bones due to differences in flight mode, hunting style, body size and dietary choices. Cubo and Casinos [25] examined the relationship of several cross-sectional geometric parameters with body size and estimated that the humerus has the largest CA, I_max_, and J for all body sizes. Main and Biewener [19] integrated cross-sectional geometry of hindlimb elements with strain gauge data collected during emu locomotion. The femur and tibiotarsus of the emu were found to exhibit circular cross sections (I_max_/I_min_ ratio near 1), a shape that is better at resisting torsional loads [19]. Habib and Ruff [26] calculated femoral to humeral torsional strength ratios from cross sections to investigate mechanical loading on avian limb bones relative to locomotion within 15 species of birds. The study included three birds of prey (Golden Eagle, Eurasian Kestrel, and Barn Owl) and found that, of the three species, the eagle exhibited the greatest femoral strength in torsion, which may relate to body size, typical prey size, and prey-capture technique [26]. Simons et al. [27] analyzed the cross-sectional geometry of forelimb elements within pelecaniform birds to examine whether shape correlated with mechanical loading patterns and found that there are cross-sectional differences between birds using different flight modes. Specifically, pelecaniforms that utilize soaring as a primary flight mode had wing elements with a circular cross section and higher polar moment of area than birds that primarily flap or flap-glide. Soaring birds tend to have a large broad wing shape and it may be that the long secondary flight feathers are placing relatively large torsional loads on wing skeleton [27].

### Bone Microstructure

Avian cortical bone is composed of predominantly primary osteons formed around primary vascular canals [28], [29]. These vascular canals can be classified into four categories based on their orientation relative to the external surface of the bone section: circular (parallel to external surface), radial (orthogonal to external surface), longitudinal (parallel to long axis of bone), and oblique (all others) [28], [30], [31]. De Margerie [31] developed a method to quantify the proportion of circular canals (Laminarity Index = # circular canals/# total canals), and found that in at least one species, the mallard, bones that are expected to experience high torsional loads, such as the humerus, ulna, and femur, exhibited a high Laminarity Index (LI). Therefore, de Margerie proposed that a bone with microstructure consisting of a large proportion of circular canals (forming laminar bone) is better at resisting the shear stresses that occur at the bone tissue level in response to torsional loading [31].

Additional research has continued to investigate how the degree of laminarity in bone relates to function in avian long bones. Skedros and Hunt [32] investigated regional variations of predominant collagen fiber orientations in the turkey ulna, finding a correlation with Laminarity Index. De Margerie et al., [33] calculated the degree of laminarity, along with other shape and microstructural features (bone wall thickness, circularity, and collagen fiber orientation), of long bone cross sections for a larger sample of birds (22 species), and found that torsion-resisting features (i.e., high laminarity) were generally found in the humerus, ulna, and femur, suggesting torsional loads may be one principal determinant in the structural makeup of these avian long bones. Specifically, the members of Accipitridae and Strigiformes included in the study were among those taxa with the most torsion-resisting features (shape and microstructure) in the humerus, ulna, and femur. The authors also suggest that bones with low laminarity may be better at resisting bending loads [33]. Additionally, Simons and O’Connor [34] investigated the Laminarity Index of avian wing elements in regards to presumed mechanical loading based on wing shape and flight style. The results indicated significant differences in laminarity between the flight modes as expected based on the different mechanical loading placed on the wing bones due to wing shape and usage. In general, higher laminarity is found in the wing elements of birds that have a broad wing shape (such as static soaring birds) as opposed to those that have a long narrow wing [33], [34].

Mechanical loading causes strain, or deformation of the bone, and can stimulate secondary (Haversian) remodeling of bone tissue [28], [29], [35], [36]. In addition, the medullary bone found within the long bones of egg-laying females is the internal mineral reservoir used for egg-shell calcification [37]. In this study, we exclude secondary and medullary bone and focus on the primary vascular canal structure to allow for the investigation of microstructural features that may be pre-adapted to resist the mechanical loads associated with certain flight and hunting behaviors.

Parameters quantifying both the cross-sectional shape and aspects of the microstructure have been used to estimate what types of load a bone experiences during locomotion [23], [25], [27], [31], [34], [38], [39]. Most relevant to this study is that both the cross-sectional geometry and degree of laminarity have been found to vary in a predictable way with differences in avian locomotion. The femur has been found to have a greater strength in torsion in an eagle that specializes on large prey than in a falcon or owl, both specializing on small prey [26]. Wing elements have been found to have a more circular cross-sectional shape and higher relative polar moment of area in birds that soar, as opposed to those that flap or flap-glide [27]. Results from bone microstructure studies generally concur with those of cross-sectional geometry and find that the femur, humerus, and ulna exhibit a highly laminar structure and that laminarity is especially high in wing bones of birds with broad wing shapes [31], [33], [34].

Based on these previous findings and the documented differences in hunting style, foot morphology, and primary flight mode between the Red-tailed Hawk and Great Horned Owl, we have developed two specific aims for this study: (1) To characterize the cross-sectional geometry and bone microstructure of limb bones and identify patterns that may be common to these two species, and (2) To investigate whether or not there are differences in these parameters between species. Are the fine-grained differences in hunting and flight behavior between the two species reflected in either the cross-sectional shape or microstructure of the skeletal elements? To address these aims, we calculated the cross-sectional geometric parameters and degree of laminarity for six limb bones: humerus, ulna, femur, tibiotarsus, hindlimb digit one phalanx one (D1P1), and hindlimb digit three phalanx three (D3P3). We chose to sample the phalanx adjacent to the talon from digits one and three due to the consistent posterior and anterior position (respectively) of these two digits in both species. Differences were investigated among bones with species combined and between species. We developed a series of predictions for each specific aim.

First, we wanted to characterize the general patterns of laminarity and cross-sectional shape of the limb elements. We predicted that the wing elements (humerus, ulna) and femur would exhibit high laminarity, high polar moment of area (J), and an I_max_/I_min_ ratio near one (circular cross section) as suggested by previous research done by de Margerie [31] and due to the torsional loads experienced by these elements [17]–[19]. We also predicted that the distal hindlimb elements (tibiotarsus, D1P1, and D3P3) in both species would exhibit low laminarity, a more elliptically shaped cross section (a shape that is better suited to resist bending in a specific direction), and high relative cortical area. These elements may experience predominantly bending and compressional loads as opposed to torsion [33].

Second, we wanted to investigate whether or not there are differences in microstructure and cross-sectional shape of limb elements between the two species. We predicted that the femur and tibiotarsus of the Great Horned Owl would exhibit higher relative cortical area than the Red-tailed Hawk due to their more vertical descent onto prey during capture [12]. Conversely, we predicted that the femur and tibiotarsus of the Red-tailed Hawk would exhibit more elliptical cross sections due to their more horizontal approach to prey and horizontal orientation of extended hindlimbs, suggesting these elements may be experiencing more bending loads. We predicted that the phalanges of the Great Horned Owl would exhibit a more elliptical cross-sectional shape and lower laminarity than the Red-tailed Hawk, suggesting greater resistance to bending loads due to the stronger grip force of the owl and the position of the digits during prey capture [9]. We predicted that the humerus and ulna of the Red-tailed Hawk would be more circular in cross section and exhibit a higher polar moment of area and laminarity than the Great Horned Owl, due to its use of static soaring as a primary flight mode. Soaring birds have been shown to exhibit high polar moments of area and more circular cross sections in wing elements, whereas flapping birds exhibit more elliptically shaped wing bone cross sections [27].

## Materials and Methods

This study used a sample of twelve specimens, six Red-tailed Hawks (*Buteo jamaicensis*) and six Great Horned Owls (*Bubo virginianus*). These two birds of prey were chosen based on known differences in flight behavior, foot morphology, and hunting/prey capture behavior. The specimens were obtained from the Arizona Game and Fish rehabilitation center and were preserved frozen. Specific age of each specimen was not available, but all specimens were determined to be adults based on plumage patterns. Sexual dimorphism is present in both species, with the female being about 20–25% larger than the male [40]. This study used both males and females based on availability. The sex of the specimens was determined by internal dissection. The mass of each bird was previously recorded at time of death. Wingspan was measured for each individual. See Table 1 for sex, mass, and wingspan data.

**Table 1:** Wingspan, mass, and sex for each individual used in this study.

Species/MWU #	Wing Span (cm)	Mass (kg)	Sex
RTH 155	128	0.7	F
RTH 156	96.8	0.69	F
RTH 210	92	1.01	M
RTH 158	121.5	0.83	F
RTH 175	115	0.92	F
RTH 211	89	0.61	M
GHO 136	120	1.25	F
GHO 212	113.5	0.96	F
GHO 213	107	1.12	M
GHO 11	105	0.95	F
GHO 137	NA	0.64	F
GHO 138	104.5	0.64	F

MWU, Midwestern University; RTH, Red-tailed Hawk; GHO, Great Horned Owl; NA, not available (wings could not be fully extended for adequate measurement).

Six limb bones were sampled: the humerus and ulna from the forelimb and the femur, tibiotarsus, D1P1, and D3P3 from the hindlimb. Previous studies have investigated both degree of laminarity and bone cross-sectional geometry of these bones in other species [25]–[27], [31]–[34], but no study has examined both these sets of parameters in all these elements in a sample of similarly sized birds of prey that differ in both flight and hunting behavior. The digit orientation differs between the Red-tailed Hawk and Great Horned Owl: the Red-tailed Hawks are zygodactylous and Great Horned Owls are anisodactylous. Digits one and three were chosen for this study because of their consistent posterior and anterior position (respectively). The phalanx adjacent to the talon on each digit was sampled (D1P1, D3P3). The usage of talons plays a considerable role in prey capture and manipulation [10], and the adjacent phalanx may show morphology that reflects differences in talon use.

### Histological Preparation

Elements were sampled from the right-side fore and hindlimb of each individual. The total length of each bone was measured using digital calipers. A 37 millimeter section surrounding the midshaft was marked (to maintain orientation) and exised. Any marrow present was rinsed away with a stream of water. Due to their small size, the phalanges were left whole. The bone segments were fixed in formalin (10% buffered neutral; two changes at 24 hours each), dehydrated in a graded ethanol series (70, 80, 95, and 100%; two changes at 24 hours each), and cleared in Histoclear (four hours). The bone segments were embedded using Osteo-Bed resin (a two-part methyl methacrylate-based material, Polysciences, Inc.). Polymerization took up to 24 hours (in a water bath at 32°C). The embedding protocol was modified from An and Martin [41] and Simons et al. [27].

After polymerization, a 1.2 mm thick section was cut from each block using a Buehler Isomet 1000 digital low speed diamond-blade saw. The sections were adhered to a plexiglass acrylic slide (Professional Plastics) using clear two-ton epoxy (Devcon). Each section was ground to a thickness of 100 µm and polished using a series of CarbiMet2/MicroCut abrasive grinding papers (grit values 320, 600, 800, and 1200, Buehler) on a Metaserv 2000 variable speed grinder-polisher (Buehler). Thickness was measured with a ±0.01 mm micrometer (Mitutuyo).

### Image Analysis

A series of images were taken from each specimen with a 4X objective using an AxioCam MRc5 digital camera (ZEISS) attached to an Olympus light microscope. The images were stitched together into a composite image using Axiovision (ZEISS) software. The complete histological images are freely accessible as interactive digital slides on the Paleohistology Repository: http://paleohistology.appspot.com [42]. Each cross section was divided into four quadrants based on orientation. The quadrants were different for forelimb (dorsal, ventral, caudal, cranial) and proximal hindlimb (caudal, cranial, medial, lateral) bones. In each quadrant, a 500 µm by 1000 µm sample area was selected between the external and internal surface of the bone. Within each sample area, the primary vascular canals were categorized into one of four categories; circular, oblique, radial and longitudinal, based on their orientation relative to the outside surface of the bone, following de Margerie [31]. Secondary osteons and inner and outer circumferential lamellar bone were not used. Laminarity was calculated by taking the ratio of circular canals to total canals for each sample. The four quadrants were averaged to represent the laminarity of the whole section. Due to the small size of the phalanges, the primary vascular canals were categorized for the entire cross section. Each Laminarity Index was arcsin transformed for normality.

To examine the cross-sectional geometry, the images were converted to black and white in Adobe Photoshop. A selection of the exterior and interior surface of the bone was made. Using the paint tool, the bone was painted white and the remainder of the photo was painted black. MomentMacroJ version 1.3 (www.hopkinsmedicine.org/fae/mmacro.htm) for Image J version 1.46 (NIH) was used to calculate the following cross-sectional geometrical parameters: second moment of area in the maximum (I_max_) and minimum (I_min_) direction, cortical area (CA) and total cross-sectional area (TA). Polar moment of area (J) was calculated by adding the second moment of area in the maximum (I_max_) and minimum (I_min_) directions. The product of total bone length (L) and body mass (M) was used to standardize J. Second moments of area, polar moment of area, and the product of bone length and body mass were log_10_ transformed for normality before the following ratios were calculated; I_max_/I_min_ and J/(L*M). CA/TA was arcsin transformed for normality.

### Statistical Analysis

ANOVA with *post hoc* Bonferroni corrected multiple comparisons was used to test for differences among bones. Student’s t-tests were used to test for differences between species. Despite transforming the data, some variables were not normally distributed or exhibited heteroscedasticity (I_max_/I_min_ and J/(L*M)); therefore, non-parametric analyses (Kruskal-Wallis and Mann-Whitney tests) were used for these variables. To control group-wide type-I error during the multiple t-tests between species, a sequential Bonferroni adjustment was applied to each bone [43]. For the four tests between species for each bone (LI, J/(L*M), I_max_/I_min_, and CA/TA) the Bonferroni-adjusted accepted alpha levels were: 0.125, 0.167, 0.25, and 0.5.

## Results

**Figure 1:**
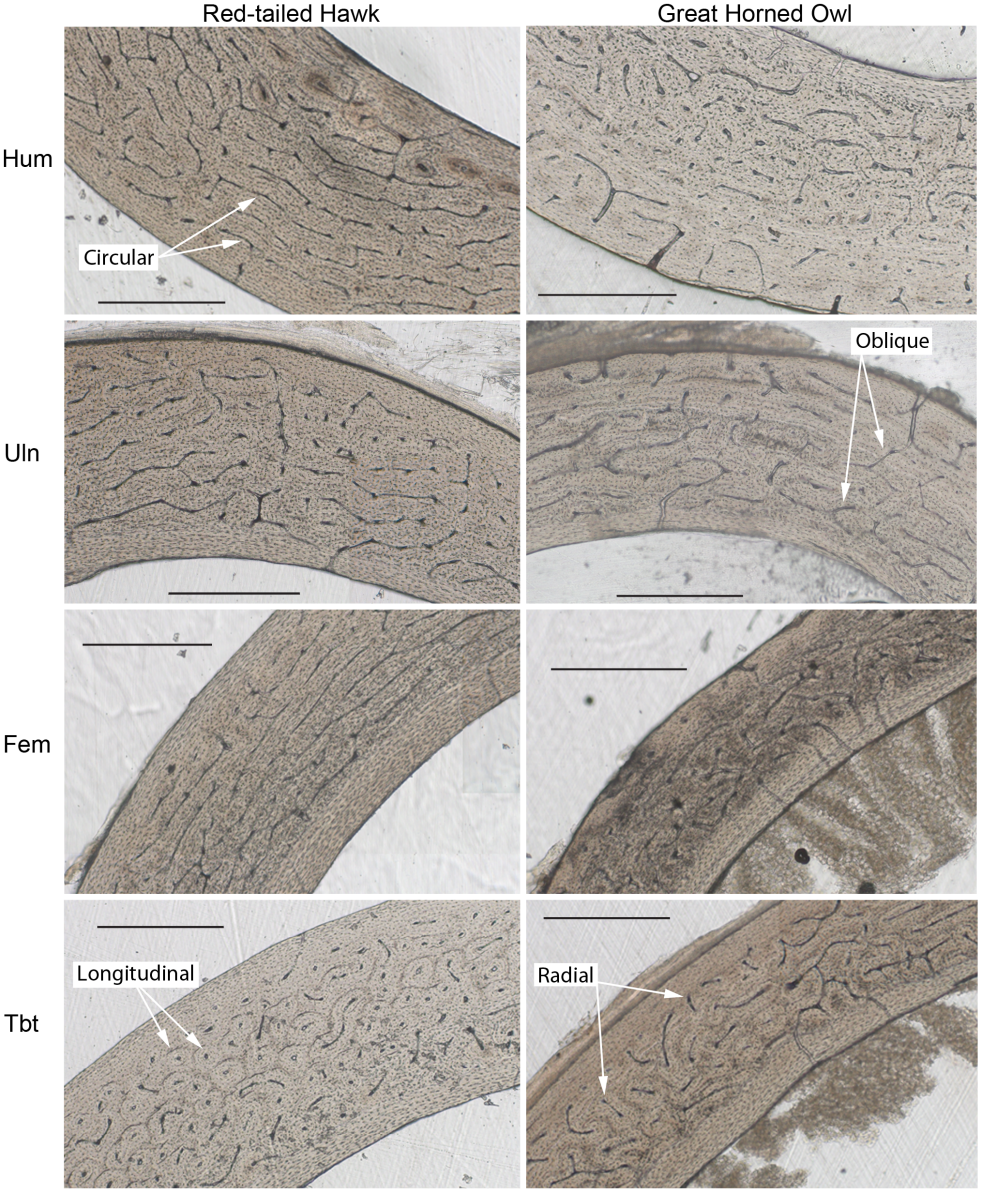
Histological comparison of the humerus (Hum), ulna (Uln), femur (Fem), and tibiotarsus (Tbt) of a Red-tailed Hawk (MWU #175) and Great Horned Owl (MWU #11). Each scale bar equals 500 µm. Examples of each of the four types of primary vascular canal orientations (circular, longitudinal, oblique, radial) are indicated by arrows.

**Figure 2:**
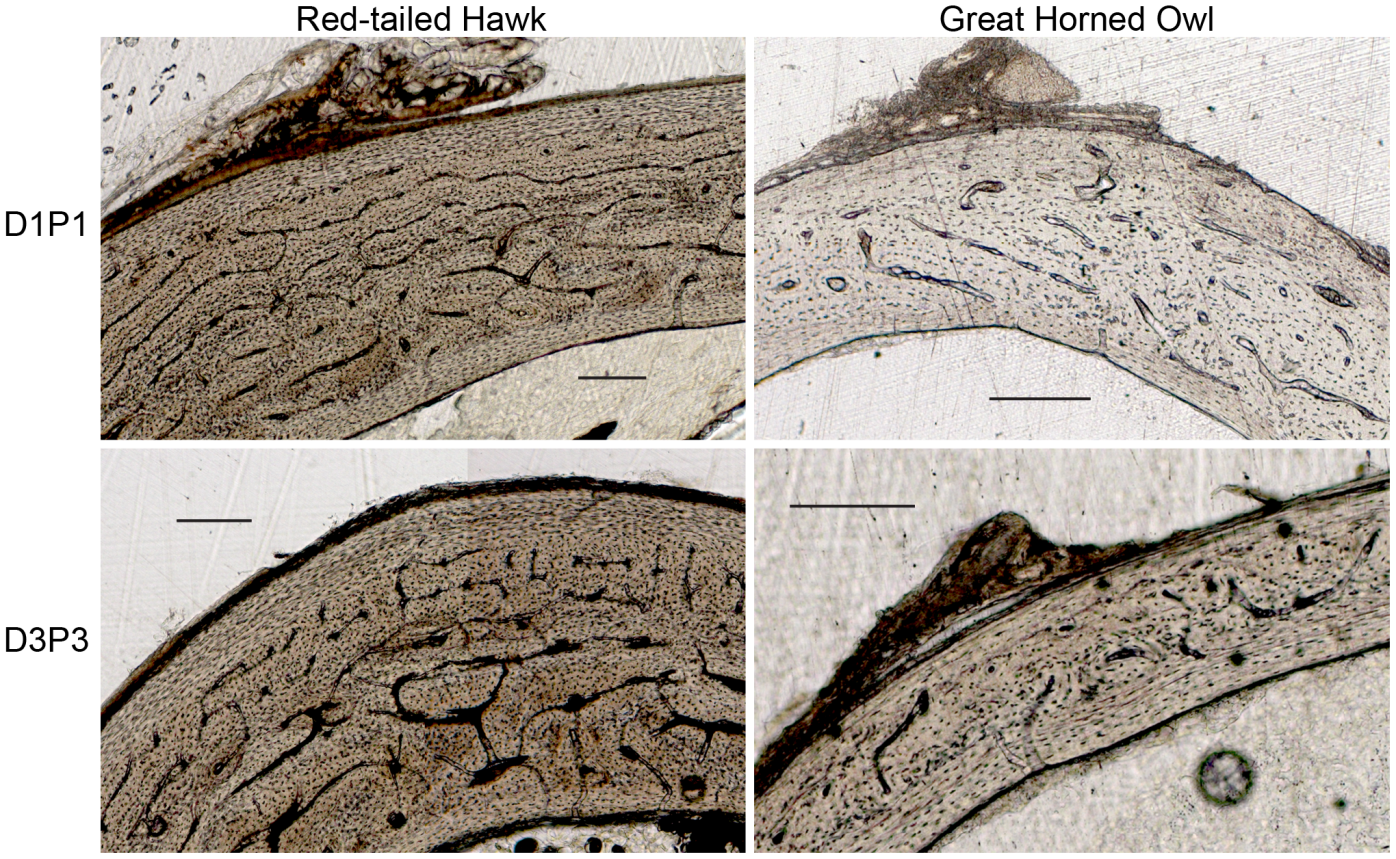
Histological comparison of digit one phalanx one (D1P1) and digit three phalanx three (D3P3) of a Red-tailed Hawk (MWU #175) and Great Horned Owl (MWU #11). Each scale bar equals 200 µm.

**Figure 3:**
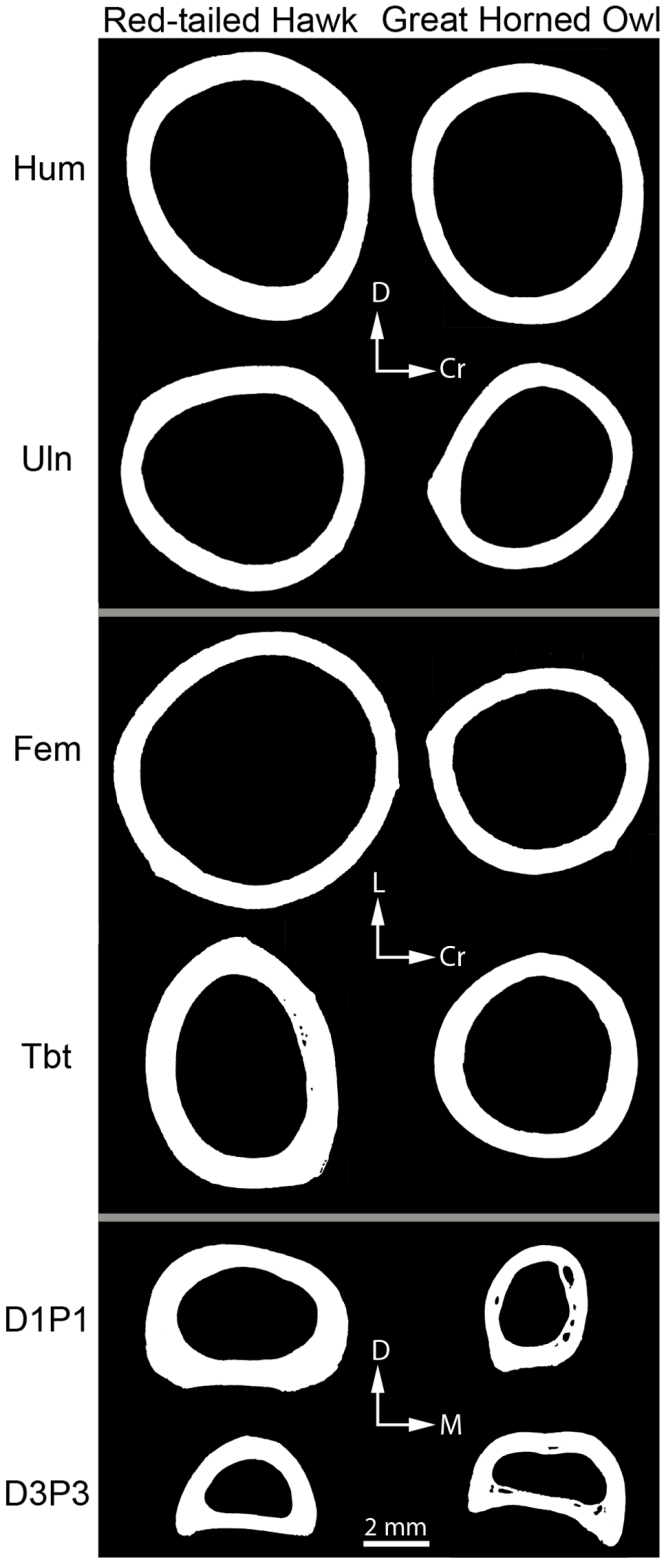
Representative cross sections of the humerus (Hum), ulna (Uln), femur (Fem), tibiotarsus (Tbt), digit one phalanx one (D1P1) and digit three phalanx three (D3P3) of a Red-tailed Hawk (MWU #155) and Great Horned Owl (MWU #213). Histological images were converted to black and white. Orientation: Dorsal (D), Cranial (Cr), Lateral (L), Medial (M). Scale bar is for all sections.

Significant differences were found among elements when species were combined and between species for both microstructural and cross-sectional geometric parameters. Results are shown in Tables 2, 3, and 4. Figures 1 and 2 show examples of microstructure and Figure 3 shows cross-sectional shape for each element of both species. Raw data can be found in Table S1.

**Table 2:** Means and standard deviations (SD) of raw data for microstructural and cross-sectional variables.

		Humerus	Ulna	Femur	Tibiotarsus	Digit 1, Phal. 1	Digit 3, Phal. 3
LI	RTH	0.40 (0.08)	0.36 (0.06)	0.45 (0.06)	0.13 (0.05)	0.40 (0.11)	0.31 (0.06)
	GHO	0.33 (0.08)	0.36 (0.07)	0.30 (0.04)	0.21 (0.05)	0.31 (0.05)	0.27 (0.07)
J/(L*M)	RTH	2.01 (0.52)	0.82 (0.47)	2.41 (0.57)	1.06 (0.23)	2.10 (0.72)	0.58 (0.18)
	GHO	1.52 (0.38)	0.52 (0.18)	1.29 (0.30)	0.78 (0.28)	0.67 (0.23)	1.09 (0.45)
I_max_/I_min_	RTH	1.28 (0.08)	1.09 (0.06)	1.18 (0.05)	1.50 (0.14)	2.44 (0.34)	2.19 (0.25)
	GHO	1.40 (0.07)	1.34 (0.08)	1.20 (0.09)	1.12 (0.03)	1.67 (0.15)	2.75 (0.32)
CA/TA	RTH	0.40 (0.03)	0.40 (0.03)	0.33 (0.03)	0.48 (0.06)	0.59 (0.06)	0.67 (0.10)
	GHO	0.38 (0.03)	0.40 (0.04)	0.39 (0.05)	0.44 (0.04)	0.54 (0.09)	0.59 (0.13)

LI, laminarity index; J/(L*M), polar moment of area standardized to bone length × body mass; I_max_/I_min_, ratio of maximum to minimum second moment of area; CA/TA, cortical area to total area; RTH, Red-tailed Hawk; GHO, Great Horned Owl.

**Table 3:** *p*-values from ANOVA (overall) and Bonferroni-corrected post hoc multiple comparisons to test for differences among bones.

	LI	J/(L*M)	I_max_/I_min_	CA/TA
Overall	**<0.001**	**<0.001**	**<0.001**	**<0.001**
Humerus - ulna	1.000	**0.003**	1.000	1.000
Humerus - femur	1.000	1.000	1.000	1.000
Humerus - tibiotarsus	**<0.001**	0.150	1.000	0.353
Humerus - D1P1	1.000	1.000	0.053	**<0.001**
Humerus - D3P3	0.617	**0.012**	**<0.001**	**<0.001**
Ulna - femur	1.000	**0.002**	1.000	1.000
Ulna - tibiotarsus	**<0.001**	1.000	1.000	0.608
Ulna - D1P1	1.000	0.392	**0.004**	**<0.001**
Ulna - D3P3	0.764	1.000	**<0.001**	**<0.001**
Femur - tibiotarsus	**<0.001**	0.087	1.000	**0.013**
Femur - D1P1	1.000	1.000	**<0.001**	**<0.001**
Femur - D3P3	0.247	**0.006**	**<0.001**	**<0.001**
Tibiotarsus - D1P1	**<0.001**	1.000	**0.014**	**0.014**
Tibiotarsus - D3P3	**<0.001**	1.000	**<0.001**	**<0.001**
D1P1 - D3P3	1.000	0.897	1.000	0.247

Significant *p*-values indicated by bold type. LI, laminarity index; J/(L*M), polar moment of area standardized to bone length × body mass; I_max_/I_min_, ratio of maximum to minimum second moment of area; CA/TA, cortical area to total area; D1P1, Digit 1 phalanx 1; D3P3, Digit 3 phalanx 3.

**Table 4:** *p*-values from Student’s t-test for differences between species.

	LI	J/(L*M)	I_max_/I_min_	CA/TA
Humerus	0.119	0.070	**0.024**	0.164
Ulna	1.000	0.352	**0.001**	0.638
Femur	**<0.001**	**0.002**	0.359	**0.026**
Tibiotarsus	**0.016**	0.082	**0.002**	0.117
D1P1	0.082	**<0.001**	0.896	0.304
D3P3	0.337	0.019	0.109	0.688

Significant *p*-values indicated by bold type. Bonferroni-adjusted accepted alpha levels for each bone: 0.125, 0.167, 0.25, 0.5. LI, laminarity index; J/(L*M), polar moment of area standardized to bone length × body mass; I_max_/I_min_, ratio of maximum to minimum second moment of area; CA/TA, cortical area to total area; D1P1, Digit 1 phalanx 1; D3P3, Digit 3 phalanx 3.

**Figure 4:**
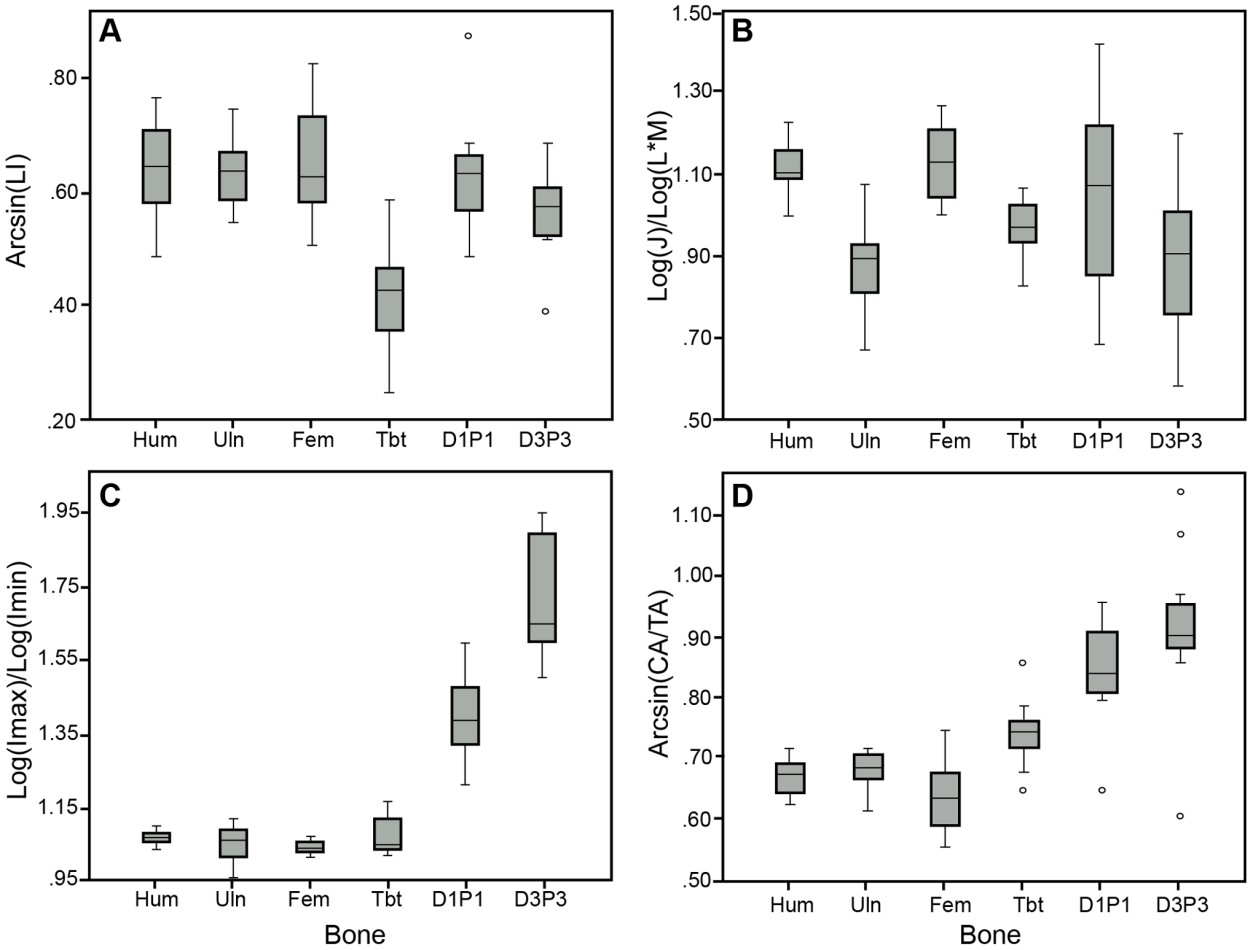
Box plots of variation in measured parameters among bones with species combined: humerus (Hum), ulna (Uln), femur (Fem), tibiotarsus (Tbt), digit one phalanx one (D1P1), and digit three phalanx three (D3P3). A) Arcsin transformed Laminarity Index (Arcsin(LI)), B) Log transformed relative polar moment of area (Log(J)/Log(L*M)), C) Ratio of log transformed maximum to minimum second moment of area (Log(I_max_)/(I_min_)), D) Arcsin transformed relative cortical area (Arcsin(CA/TA)). Median line shown in boxes. Whiskers show 10^th^ and 90^th^ percentiles. Outliers indicated by small circles. Significant results are as follows: The tibiotarsus had lower LI than all other bones and more relative cortical area than the femur. The humerus and femur showed larger relative polar moments of area than the ulna and D3P3. D1P1 and D3P3 were more elliptical and had more relative cortical area than other bones.

With species combined, one-way ANOVA (or Kruskal-Wallis) tests indicated the following significant differences among elements (Table 3). The microstructure of the humerus, ulna, femur, D1P1, and D3P3 were significantly more laminar than the tibiotarsus (p<0.001) (Figure 4A). The relative polar moments of area (J/(L*M)) of the humerus and femur were significantly greater than the ulna (p = 0.003, p = 0.002) and D3P3 (p = 0.012, p = 0.006) (Figure 4B).The cross sections of D1P1 and D3P3 were significantly more elliptical than the ulna (p = 0.004, p<0.001), femur (p<0.001, p<0.001), and tibiotarsus (p = 0.014, p<0.001). In addition, the cross section of D3P3 was significantly more elliptical than the humerus (p<0.001) (Figure 4C). D1P1 and D3P3 exhibited higher CA/TA than the humerus (p<0.001, p<0.001), ulna (p<0.001, p<0.001), femur (p<0.001, p<0.001), and tibiotarsus (p = 0.014, p<0.001). In addition, the tibiotarsus exhibited higher CA/TA than the femur (p = 0.013) (Figure 4D).

**Figure 5:**
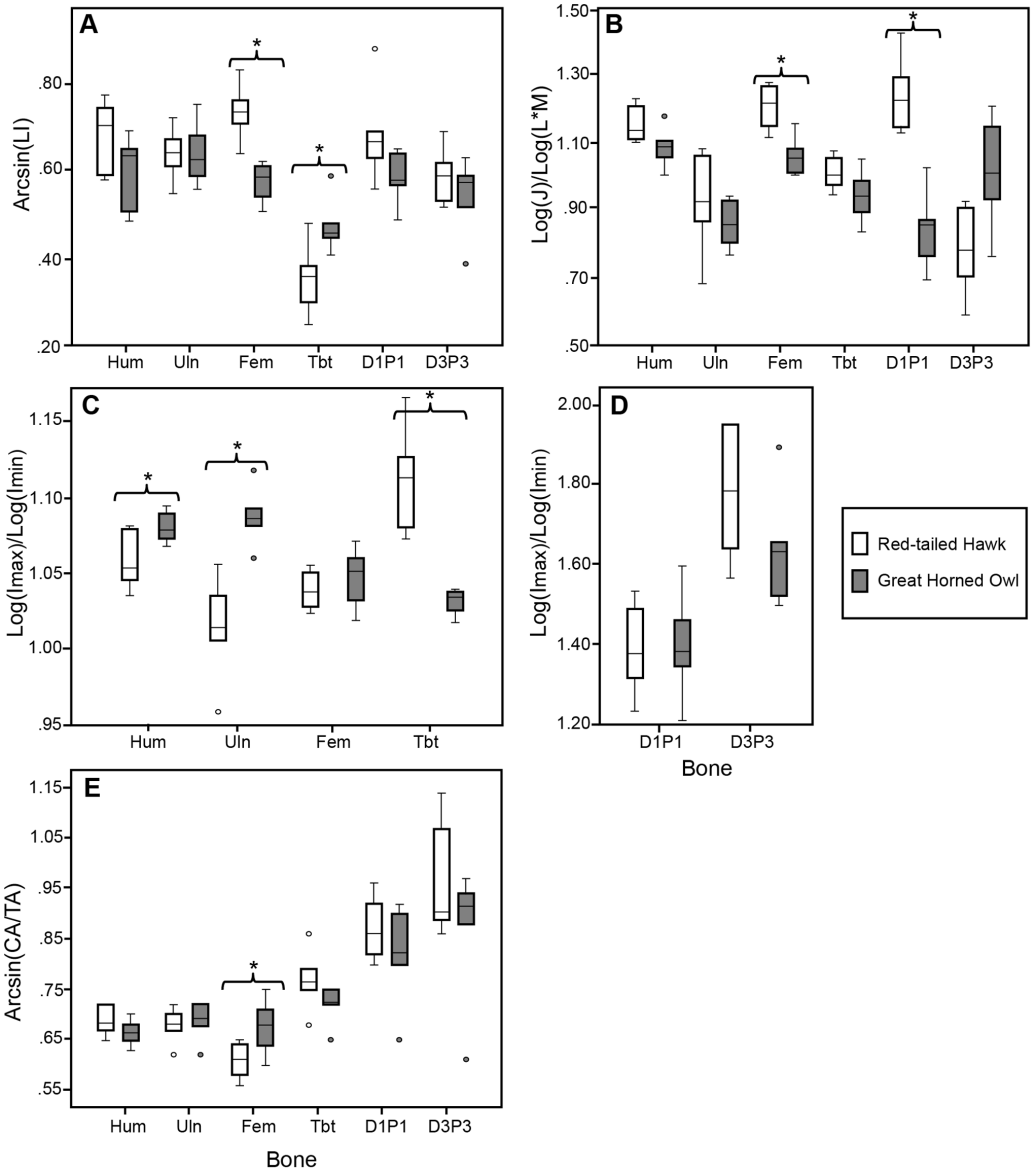
Box plots of variation in measured parameters and student’s t-test results between Red-tailed Hawk (white boxes) and Great Horned Owl (gray boxes) for humerus (Hum), ulna (Uln), femur (Fem), tibiotarsus (Tbt), digit one phalanx one (D1P1), digit three phalanx three (D3P3). A) Arcsin transformed Laminarity Index (Arcsin(LI)), B) Log transformed relative polar moment of area (Log(J)/Log(L*M)), C) Ratio of log transformed maximum to minimum second moment of area (Log(I_max_)/(I_min_)) for proximal bones, D) Log(I_max_)/(I_min_) for phalanges, E) Arcsin transformed relative cortical area (Arcsin(CA/TA)). Median line shown in boxes. Whiskers show 10^th^ and 90^th^ percentiles. Outliers indicated by small circles. Significant differences indicated by bracket and asterisks. Significant results are as follows: The femur of the RTH had larger LI, larger relative polar moment of area, and lower relative cortical area than the GHO. The humerus and ulna of the GHO were more elliptical than the RTH. The tibiotarsus of the GHO had larger LI and was more elliptical than the RTH. The D1P1 of the RTH had larger relative polar moment of area than the GHO.

T-tests (or Mann-Whitney) between species indicated that the femur of the RTH was significantly more laminar than the GHO (p<0.001) and the tibiotarsus of the GHO was significantly more laminar than the RTH (p = 0.016) (Table 4, Figure 5A). For the cross-sectional geometric parameters, the RTH femur and D1P1 exhibited higher relative polar moment of area (J/(L*M)) than the GHO (p = 0.002, p<0.001) (Figure 5B). The RTH tibiotarsus was more elliptical than the GHO (p = 0.002). The GHO humerus and ulna were more elliptical than the RTH (p = 0.024, p = 0.001) (Figure 5C). The GHO femur exhibited higher CA/TA than the RTH (p = 0.026) (Figure 5E).

## Discussion

### Combined species patterns

The first specific aim of this study was to characterize the bone microstructure and cross-sectional shape for each bone: humerus, ulna, femur, tibiotarsus, D1P1, and D3P3 with the two species combined. Combining the species in these analyses allows for examination of features common to these two species or perhaps to birds of prey in general.

#### Cross-sectional Geometry

We predicted that the wing elements (humerus, ulna) and femur in both species would exhibit an I_max_/I_min_ ratio near one and a high polar moment of area (J). Bones that are better resistant to torsional loading are expected to be more circular in shape and exhibit high polar moments of area. Previous research has shown that the humerus and femur experience torsional loading during locomotion [17]–[19]. As predicted, the humerus, ulna, and femur exhibited an I_max_/I_min_ ratio close to one, and the humerus and femur exhibited a high relative polar moment of area (Figure 4B, C). This supports the results of previous studies in which the humerus and femur, at least, are demonstrated to be better resistant to torsional loads.

The distal hindlimb elements (tibiotarsus, D1P1, D3P3) in both species were predicted to exhibit a more elliptically shaped cross section, implying a shape that is better suited to resist bending in a specific plane. Previous research has suggested that distal hindlimb elements may be experiencing predominantly bending and compressional loads as opposed to torsion [19], [33], [44]. The results indicated that the cross sections of D1P1 and D3P3 were more elliptical than nearly all other bones (Figure 4C). The elliptical orientation suggests that the phalanges may be better resistant to bending in a specific plane. An alternative explanation is that the phalanges have an elliptical cross-sectional shape to accommodate the large tendons that run along the dorsal and plantar surfaces. Most of the phalanges are elliptical in the medial-lateral plane, with one exception (see discussion below). Counter to prediction, the tibiotarsus did not have an elliptical cross-sectional shape when species were combined, but did show an interesting difference between species (see below). This implies that the cross-sectional shape of the tibiotarsus may not be common to all raptors, but may be more dependent on usage.

Another prediction states that the distal hindlimb elements (tibiotarsus, D1P1, D3P3) in both species would exhibit high relative cortical area (CA/TA) because these hindlimb elements are expected to be experiencing compression loads. The phalanges (D1P1 and D3P3) did exhibit higher relative cortical area than the humerus, ulna, femur, and tibiotarsus (Figure 4D). In addition, the tibiotarsus exhibited higher relative cortical area than the femur (Figure 4D). These results were expected since the tibiotarsus is predicted to experience axial compression due to its vertical orientation during walking [19]. The feet of these birds of prey are used for prey capture and dispatch [9]. Each digit is tipped with a large claw, the talon. The main muscles that act to flex the talons and digits originate from the tibiotarsus and the tendons of these muscles pass distally to attach to the base of the bony core supporting the keratinous talon sheath [11]. The tendons are held close to the plantar surface of each phalanx by a tough tendon sheath [11], [45], [46]. It seems likely that the pull of tendons on the talon during the grasping and holding of struggling prey is placing the adjacent phalanx under compressional and bending loads, as shown by the relatively high CA/TA and elliptical cross-sectional shape of these bones.

#### Microstructure

We predicted that the wing elements (humerus, ulna) and femur in both species would exhibit high laminarity as suggested by previous research and due to the torsional loads experienced by these elements [17]–[19], [31], [33], [34]. As predicted, the humerus, ulna, and femur exhibited significantly higher laminarity than the tibiotarsus (Figure 4A). This result is consistent with the cross-sectional geometry findings above and further supports the hypothesis that this specific microstructure is an adaptation to resist the torsional loads placed on these elements.

We also predicted that the distal hindlimb elements (tibiotarsus, D1P1, D3P3) in both species would exhibit low laminarity. As expected, the laminarity of the tibiotarsus was low, but counter to prediction, the laminarity of D1P1 and D3P3 was higher than the tibiotarsus and not significantly different from the humerus, ulna, and femur (Figure 4A). This contradicts the results of the cross-sectional geometry of the phalanges discussed above and suggests that the microstructure and cross-sectional geometry signals may not always agree. Degree of laminarity of avian foot elements is not as well studied as laminarity in the more proximal elements, but it is not completely unknown. Laminarity of digit three phalanx one (D3P1) was observed to be generally low in birds of prey by de Margerie et al. [33], with the exception of the Common Barn Owl (*Tyto alba*). High laminarity in phalanges may suggest that the foot elements in these species may be experiencing relatively large or repetitive torsional loads during foot grasping.

Although the Laminarity Index values in this study would all be classified as low (<0.5, [31]; Tables 2, S1), the pattern of laminarity in the proximal limb elements is as predicted based on previous research. Further investigation of specific laminarity indices of limb element of more avian species may help determine what should be considered low and high laminarity. In addition, this study has shown that there are differences in laminarity present among limb elements in these two species. A previous study of only forelimb elements found no significant differences in LI among bones and postulated that microstructure may be strictly genetically determined [34]. The inclusion of hindlimb elements here shows that the tibiotarsus, at least, exhibits a different microstructure than other elements.

### Between species patterns

The second specific aim of this study was to investigate differences in microstructure and cross-sectional geometry between the two species in this study. The RTH and GHO are similar-sized birds of prey. No differences were found between mass and measured wingspan between the two groups (p = 0.304, p = 0.453). Both species are sit-and-wait predators that hunt with their hindlimbs, but have different specific prey capture behaviors. The GHO descends on prey and uses its stronger grip force constrict and immobilize prey. The RTH approaches from the horizontal and strikes with the hindlimbs extended and uses their large hallux to grasp prey [9].

#### Cross-sectional Geometry

Our first prediction was that the femur and tibiotarsus of the GHO would exhibit cross-sectional properties that imply greater resistance to compression (high CA/TA) than the RTH due to their vertical descent onto prey during capture [12]. The femur of the GHO did exhibit larger relative cortical area than the RTH (Figure 5E). In the tibiotarsus, however, a trend in the data seemed to suggest a slightly larger relative cortical area in the RTH. These results do not support the prediction that the two elements of the GHO would have a shape more optimized to resist compression. Since so few significant differences were found between species for relative cortical area, this suggests that amount of cortical area may either be a genetically determined trait common to raptors, or an example of convergence between these two species.

We also predicted that the femur and tibiotarsus of the RTH would exhibit cross-sectional properties that imply greater resistance to bending than the GHO due to their more horizontal approach to prey and horizontal orientation of extended hindlimbs. Indeed, the tibiotarsus of the RTH was significantly more elliptical than the GHO in the medial-lateral plane, suggesting that the tibiotarsus of the RTH may be experiencing more bending loads in this plane (Figure 5C). Since the GHO displayed a more circularly shaped tibiotarsus, it may be better suited to resist torsional loads or bending in multiple directions rather than in one specific plane. No significant differences were found in ellipticality of the femur between the two species. The femora of both species are very circular in cross section. The femur of RTH, however, exhibited a higher relative polar moment of area than the GHO (Figure 5B). This pattern was reflected in the microstructure (see below). The RTH femur, therefore, has both a microstructure and cross-sectional shape that indicate a higher resistance to torsional loads than the GHO. The larger mass of the prey may cause larger torsional loads on the proximal limb element (femur) in the RTH [6], [9], [11], [14]. In addition, the RTH is more likely to pursue an escaped prey item on the ground [15]. This ground pursuit may cause more torsional loading on the femur and potentially more regular bending loads on the tibiotarsus.

The phalanges (D1P1 and D3P3) of the GHO were predicted to exhibit a more elliptical cross-sectional shape than the RTH, suggesting greater resistance to bending loads due to the stronger grip force of the owl and the position of the digits (zygodactylous) during prey capture [9]. However, no differences were found in I_max_/I_min_ ratio between the two species for either D1P1 or D3P3 (Figure 5D). Interestingly, in D1P1, despite there being no overall difference in shape, the RTH was more elliptical in the medial-lateral plane and the GHO was more elliptical in the dorsal-plantar plane (Figure 3). In fact, the D1P1 of the GHO is the only phalanx in the study to be elliptical in the dorsal-plantar plane. It is unclear why the GHO D1P1 may have this shape. In the GHO, digit one is not the only digit facing posteriorly; digit four does as well. A more complete sampling of phalanges from each of the four digits in the future may allow for some patterns to be determined.

Although no significant differences were found between species in the ellipticality of the phalanges, the D1P1 of the RTH exhibited larger relative polar moment of area than the GHO (Figure 5B). A cross section with large polar moment of area is generally interpreted to be more resistant to torsional loads; however, this interpretation is most robust when cross sections are nearly circular (I_max_/I_min_ <1.5; [47]). The raw I_max_/I_min_ values for D1P1 were greater than 1.5 for both species (Table 2). It is possible this difference may partially be driven by differences in total bone length. Student’s t-test shows that the D1P1 of the RTH is significantly longer than the GHO (p<0.001). Digit one in the RTH is the only posteriorly directed digit, bears a talon that has been shown to be significantly larger than other raptors, and is used to grip prey [10]. Overall, these differences in foot morphology and hunting behavior begin to explain the variation found in proximal bone structure (femur and tibiotarsus), but further investigation of the cross-sectional geometry of distal hindlimb elements (additional phalanges of all digits, and tarsometatarsus) may help clarify these patterns.

In the forelimb, we predicted that the humerus and ulna of the RTH would be more circular in cross section and exhibit a higher polar moment of area than the GHO. The RTH uses static soaring as a primary flight mode and the GHO uses flap-gliding. The results showed that the RTH humerus and ulna were statistically more circular than the GHO (Figure 5C). This result supports that of a previous study that showed the wing elements of soaring birds were nearly circular in cross section [27]. The GHO humerus and ulna were more elliptical than the RTH. The larger amount of flapping in the flight mode of the GHO may place more bending loads on the wing elements. The elements are elliptical in the dorsal-ventral plane as would be expected to resist the aerodynamic loads of upstroke and downstroke [17]. There were, however, no significant differences in relative polar moment of area between species (Figure 5B). The trend in the data suggested that the RTH had slightly higher relative polar moment of area than the GHO. A larger sample size may increase those differences.

#### Microstructure

We predicted that the phalanges D1P1 and D3P3 of the GHO would exhibit a lower Laminarity Index than the RTH. The phalanges of the GHO were expected to be experiencing more bending loads than the RTH, primarily due to their use of high force grip of their talons. However, no differences were found in laminarity of D1P1 or D3P3 between species. We also predicted that the humerus and ulna of the RTH would exhibit a higher Laminarity Index than the GHO due to the RTH’s static soaring flight style. The results indicated that there were no microstructural differences in the humerus or ulna between the species. This is likely due to the fact that these two species both have large broad wings of similar aspect ratio. It has previously been shown in at least one group of birds that despite differences in cross-sectional geometry of wing bones among birds that use different flight modes, no difference is found in degree of laminarity when wings are similarly shaped [27], [34]. These results suggest that lack of differences between these two species may be due to other factors affecting bone microstructure, namely phylogenetic factors, growth, or environment, rather than mechanical function. However, although no specific predictions were made about the LI of the femur and tibiotarsus between the two species, the RTH femur was found to exhibit higher laminarity than the GHO and the GHO tibiotarsus exhibited higher laminarity than the RTH (Figure 5A). This pattern is partially reflected in the cross-sectional geometry.

In conclusion, this is the first study to investigate both the microstructure and cross-sectional shape of limb bones of two similarly sized raptors and test hypotheses based on differences in flight and hunting behavior. Both microstructure and cross-sectional geometric parameters have previously been used to estimate what types of load a bone experiences during locomotion. In this study, the results from microstructure and cross-sectional analyses generally agree, in that they correspond with predictions made and support previous research regarding the types of loads the bones may be primarily experiencing. However, in some instances the microstructure and cross-sectional parameters provide conflicting information about what types of load may be experienced. Therefore, some caution is needed when estimating biomechanical load from either bone microstructure or cross-sectional geometry. An important avenue for future research will be investigating in more species when microstructure and cross-sectional geometry signals coincide and when they are decoupled.

Another consideration is that the Red-tailed Hawk and Great Horned Owl are distantly related species. Differences observed in the cross-sectional shape and microstructure of the limb elements may simply reflect their phylogenetic relationship. To begin to test whether or not phylogeny plays a role, future studies should include additional species. For example, sampling a non-soaring accipitrid, such as *Accipiter gentilis*, could address whether the more circular humerus and ulna of the RTH are due to its soaring flight mode or are traits common to accipitrids. In addition, an owl with a relatively long tarsometatarsus, such as *Tyto alba* [11], may not have the same the higher force grip as the GHO and could be used to test whether differences observed in the hindlimb elements between the GHO and RTH are due to differences in grip strength or other aspects of prey capture behavior. The results of this type of methodology have the potential to be useful for inferring behavior in both extant taxa that may be difficult to directly observe, and extinct taxa.

## Supporting Information

Table S1 **Raw data collected from each specimen.**
(DOCX)
