# Supplementary material for: Microstructure and Cross-Sectional Shape of Limb Bones in Great Horned Owls and Red-Tailed Hawks: How Do These Features Relate to Differences in Flight and Hunting Behavior?
Source: PLoS One. 2014 Aug 27;9(8):e106094. doi: 10.1371/journal.pone.0106094 (PMC4146594; doi:10.1371/journal.pone.0106094)
Supplement: Table S1 — Raw data collected from each specimen. (DOCX) [file pone.0106094.s001.docx]

Table S1. Raw data collected from each specimen.

| MWU # | Bone | LI | I_max_ | I_min_ | J | CA | TA | Length (mm) |
| --- | --- | --- | --- | --- | --- | --- | --- | --- |
| RTH 155 | Humerus | 0.44 | 73.24 | 53.46 | 126.70 | 13.89 | 35.40 | 115.30 |
| RTH 156 | Humerus | 0.45 | 69.45 | 50.68 | 120.13 | 13.52 | 34.67 | 103.12 |
| RTH 210 | Humerus | 0.31 | 93.45 | 80.02 | 173.47 | 17.33 | 40.09 | 104.80 |
| RTH 158 | Humerus | 0.48 | 140.43 | 113.19 | 253.62 | 18.73 | 51.73 | 110.62 |
| RTH 175 | Humerus | 0.40 | 153.28 | 118.33 | 271.61 | 21.75 | 49.83 | 114.55 |
| RTH 211 | Humerus | 0.30 | 65.84 | 53.20 | 119.05 | 13.74 | 33.95 | 107.40 |
| GHO 136 | Humerus | 0.41 | 117.68 | 84.98 | 202.67 | 18.05 | 43.67 | 123.50 |
| GHO 212 | Humerus | 0.22 | 102.28 | 70.13 | 172.41 | 15.43 | 42.13 | 118.94 |
| GHO 213 | Humerus | 0.34 | 82.01 | 59.42 | 141.43 | 13.93 | 38.49 | 124.17 |
| GHO 11 | Humerus | 0.24 | 108.44 | 72.38 | 180.83 | 16.50 | 41.96 | 118.36 |
| GHO137 | Humerus | 0.37 | 64.08 | 48.99 | 113.08 | 12.13 | 35.08 | 116.49 |
| GHO 138 | Humerus | 0.37 | 97.06 | 69.96 | 167.03 | 15.91 | 40.48 | 121.00 |
| RTH 155 | Ulna | 0.43 | 76.52 | 66.01 | 142.53 | 14.67 | 37.87 | 140.92 |
| RTH 156 | Ulna | 0.38 | 26.18 | 24.53 | 50.71 | 9.38 | 21.75 | 136.69 |
| RTH 210 | Ulna | 0.34 | 42.21 | 41.17 | 83.38 | 11.68 | 28.28 | 127.32 |
| RTH 158 | Ulna | 0.33 | 13.65 | 11.88 | 25.53 | 5.73 | 16.85 | 140.07 |
| RTH 175 | Ulna | 0.39 | 82.57 | 79.95 | 162.53 | 15.70 | 40.45 | 134.43 |
| RTH 211 | Ulna | 0.27 | 30.65 | 27.24 | 57.90 | 9.67 | 23.70 | 125.86 |
| GHO 136 | Ulna | 0.31 | 34.57 | 28.32 | 62.89 | 10.17 | 24.37 | 139.52 |
| GHO 212 | Ulna | 0.31 | 36.54 | 24.95 | 61.49 | 9.82 | 24.23 | 148.72 |
| GHO 213 | Ulna | 0.28 | 26.82 | 20.29 | 47.11 | 8.52 | 21.46 | 137.33 |
| GHO 11 | Ulna | 0.40 | 55.93 | 41.20 | 97.14 | 12.95 | 29.74 | 138.15 |
| GHO137 | Ulna | 0.38 | 28.82 | 21.89 | 50.71 | 7.93 | 23.38 | 133.13 |
| GHO 138 | Ulna | 0.46 | 36.30 | 27.42 | 63.72 | 10.51 | 24.19 | 140.07 |
| RTH 155 | Femur | 0.43 | 98.90 | 83.82 | 182.73 | 14.54 | 46.64 | 85.48 |
| RTH 156 | Femur | 0.47 | 74.70 | 67.25 | 141.95 | 13.65 | 39.71 | 72.64 |
| RTH 210 | Femur | 0.46 | 71.72 | 61.14 | 132.85 | 13.36 | 37.85 | 79.80 |
| RTH 158 | Femur | 0.54 | 98.70 | 79.13 | 177.83 | 13.54 | 47.77 | 87.91 |
| RTH 175 | Femur | 0.36 | 117.64 | 103.13 | 220.78 | 17.48 | 48.31 | 88.95 |
| RTH 211 | Femur | 0.42 | 49.99 | 40.79 | 90.78 | 10.08 | 33.33 | 82.99 |
| GHO 136 | Femur | 0.26 | 59.13 | 47.48 | 106.62 | 13.30 | 31.66 | 76.04 |
| GHO 212 | Femur | 0.24 | 61.83 | 47.09 | 108.92 | 12.17 | 33.93 | 80.13 |
| GHO 213 | Femur | 0.33 | 47.61 | 42.08 | 89.69 | 11.71 | 29.82 | 77.95 |
| GHO 11 | Femur | 0.28 | 42.07 | 34.06 | 76.13 | 9.48 | 29.84 | 76.49 |
| GHO137 | Femur | 0.33 | 33.82 | 28.91 | 62.73 | 9.83 | 24.93 | 73.51 |
| GHO 138 | Femur | 0.34 | 45.03 | 41.79 | 86.83 | 12.91 | 27.61 | 74.62 |
| RTH 155 | Tibiotarsus | 0.09 | 73.05 | 45.34 | 118.39 | 15.51 | 31.03 | 121.80 |
| RTH 156 | Tibiotarsus | 0.21 | 37.19 | 22.27 | 59.47 | 12.09 | 21.18 | 108.47 |
| RTH 210 | Tibiotarsus | 0.11 | 55.84 | 41.47 | 97.31 | 13.71 | 28.90 | 109.40 |
| RTH 158 | Tibiotarsus | 0.14 | 61.64 | 38.99 | 100.64 | 12.23 | 31.14 | 119.29 |
| RTH 175 | Tibiotarsus | 0.14 | 77.38 | 57.49 | 134.88 | 15.85 | 34.34 | 114.43 |
| RTH 211 | Tibiotarsus | 0.06 | 41.60 | 29.36 | 70.96 | 11.81 | 24.51 | 113.53 |
| GHO 136 | Tibiotarsus | 0.19 | 60.07 | 52.57 | 112.65 | 14.54 | 31.52 | 117.03 |
| GHO 212 | Tibiotarsus | 0.20 | 56.44 | 49.30 | 105.74 | 13.53 | 31.09 | 119.09 |
| GHO 213 | Tibiotarsus | 0.16 | 44.55 | 41.55 | 86.10 | 12.28 | 28.16 | 188.45 |
| GHO 11 | Tibiotarsus | 0.31 | 35.38 | 31.18 | 66.56 | 9.72 | 26.41 | 116.70 |
| GHO137 | Tibiotarsus | 0.21 | 30.36 | 26.85 | 57.21 | 10.45 | 22.38 | 120.43 |
| GHO 138 | Tibiotarsus | 0.19 | 48.25 | 43.84 | 92.09 | 12.88 | 28.82 | 116.20 |
| RTH 155 | Digit 1 Phalanx 1 | 0.40 | 38.44 | 19.32 | 57.76 | 11.73 | 20.38 | 24.70 |
| RTH 156 | Digit 1 Phalanx 1 | 0.60 | 15.80 | 6.39 | 22.19 | 6.72 | 12.68 | 21.69 |
| RTH 210 | Digit 1 Phalanx 1 | 0.36 | 22.75 | 10.67 | 33.42 | 9.57 | 15.05 | 22.02 |
| RTH 158 | Digit 1 Phalanx 1 | 0.35 | 31.37 | 10.94 | 42.32 | 9.69 | 16.74 | 24.42 |
| RTH 175 | Digit 1 Phalanx 1 | 0.41 | 37.85 | 15.71 | 53.56 | 12.48 | 18.48 | 23.67 |
| RTH 211 | Digit 1 Phalanx 1 | 0.29 | 18.34 | 6.69 | 25.03 | 6.99 | 13.50 | 24.02 |
| GHO 136 | Digit 1 Phalanx 1 | 0.30 | 9.10 | 4.95 | 14.05 | 6.12 | 10.03 | 17.29 |
| GHO 212 | Digit 1 Phalanx 1 | 0.29 | 7.50 | 5.28 | 12.78 | 5.29 | 10.20 | 19.71 |
| GHO 213 | Digit 1 Phalanx 1 | 0.30 | 5.96 | 3.60 | 9.56 | 4.62 | 8.67 | 17.37 |
| GHO 11 | Digit 1 Phalanx 1 | 0.22 | 4.86 | 2.69 | 7.55 | 3.24 | 8.83 | 19.74 |
| GHO137 | Digit 1 Phalanx 1 | 0.36 | 4.44 | 2.78 | 7.22 | 4.09 | 7.48 | 15.72 |
| GHO 138 | Digit 1 Phalanx 1 | 0.35 | 7.45 | 4.44 | 11.88 | 5.81 | 9.14 | 17.54 |
| RTH 155 | Digit 3 Phalanx 3 | 0.24 | 6.73 | 3.37 | 10.10 | 5.04 | 8.04 | 18.45 |
| RTH 156 | Digit 3 Phalanx 3 | 0.40 | 2.98 | 1.19 | 4.17 | 3.21 | 5.21 | 16.48 |
| RTH 210 | Digit 3 Phalanx 3 | 0.32 | 4.81 | 2.55 | 7.36 | 5.11 | 6.67 | 17.21 |
| RTH 158 | Digit 3 Phalanx 3 | 0.34 | 6.29 | 2.65 | 8.94 | 4.50 | 7.45 | 19.24 |
| RTH 175 | Digit 3 Phalanx 3 | 0.30 | 9.12 | 3.83 | 12.96 | 7.10 | 8.64 | 17.57 |
| RTH 211 | Digit 3 Phalanx 3 | 0.26 | 4.31 | 2.12 | 6.42 | 3.83 | 6.64 | 18.67 |
| GHO 136 | Digit 3 Phalanx 3 | 0.30 | 16.87 | 6.56 | 23.43 | 7.82 | 11.51 | 20.42 |
| GHO 212 | Digit 3 Phalanx 3 | 0.29 | 18.00 | 5.85 | 23.85 | 7.40 | 11.28 | 21.63 |
| GHO 213 | Digit 3 Phalanx 3 | 0.25 | 13.17 | 4.87 | 18.05 | 6.36 | 10.34 | 19.78 |
| GHO 11 | Digit 3 Phalanx 3 | 0.15 | 7.41 | 2.88 | 10.29 | 3.27 | 9.99 | 22.70 |
| GHO137 | Digit 3 Phalanx 3 | 0.31 | 12.52 | 5.25 | 17.78 | 6.35 | 10.69 | 19.24 |
| GHO 138 | Digit 3 Phalanx 3 | 0.35 | 18.99 | 5.92 | 24.91 | 7.61 | 11.86 | 22.55 |

MWU, Midwestern University; LI, laminarity index; I_max,_ second moment of area in the maximum direction; I_min_, second moment of area in the minimum direction; J, polar moment of area; CA, cortical area; TA, total area; RTH, Red-tailed Hawk; GHO, Great Horned Owl
